# WECHAT, WERUN: A GROUP-BASED WALKING STUDY TO ENHANCE PHYSICAL ACTIVITY AMONG COMMUNITY-DWELLING OLDER ADULTS

**DOI:** 10.1093/geroni/igz038.512

**Published:** 2019-11-08

**Authors:** Yujun Liu, Margie E Lachman

**Affiliations:** 1 Brandeis University, Waltham, United States; 2 Brandeis University, Waltham, Massachusetts, United States

## Abstract

The benefits of physical activity (PA) and social engagement for older adults are well established, yet the majority of adults over age 50 in the U.S. do not engage in regular exercise. We conducted a 4-week pre-post study with a one-month follow-up using a smart phone application called WeChat WeRun to explore the mechanisms of social engagement and competition to increase PA among older adults. Participants (N=40, mean age=65.7) were randomly assigned to one of two conditions. Participants in the activity competition condition used the WeRun to track their daily walking steps and interact via text with their group members for 4 weeks. The daily walking steps were displayed to the group and the participant with the highest daily steps in the group was indicated as the champion each day. Participants in the control group only used WeRun to track their own walking steps. Outcome variables included average weekly steps, exercise self-efficacy, and social engagement. The two-way repeated measures ANOVA results revealed that participants in the competition condition had higher average weekly steps and level of social engagement at the posttest, compared to those in the control group. Post hoc tests using the Bonferroni corrections revealed that the intervention increased their average weekly steps and social engagement from pretest to post-test, while these outcomes did not change in the control group. The effects were maintained at the one-month follow up. Discussion will consider the motivational role of competition and social interactions in increasing PA among older adults.

